# Research on steel rail surface defects detection based on improved YOLOv4 network

**DOI:** 10.3389/fnbot.2023.1119896

**Published:** 2023-02-09

**Authors:** Zengzhen Mi, Ren Chen, Shanshan Zhao

**Affiliations:** College of Mechanical Engineering, Chongqing University of Technology, Chongqing, China

**Keywords:** rail defects, machine vision, defects detection, image enhancement, convolutional neural network (CNN)

## Abstract

**Introduction:**

The surface images of steel rails are extremely difficult to detect and recognize due to the presence of interference such as light changes and texture background clutter during the acquisition process.

**Methods:**

To improve the accuracy of railway defects detection, a deep learning algorithm is proposed to detect the rail defects. Aiming at the problems of inconspicuous rail defects edges, small size and background texture interference, the rail region extraction, improved Retinex image enhancement, background modeling difference, and threshold segmentation are performed sequentially to obtain the segmentation map of defects. For the classification of defects, Res2Net and CBAM attention mechanism are introduced to improve the receptive field and small target position weights. The bottom-up path enhancement structure is removed from the PANet structure to reduce the parameter redundancy and enhance the feature extraction of small targets.

**Results:**

The results show the average accuracy of rail defects detection reaches 92.68%, the recall rate reaches 92.33%, and the average detection time reaches an average of 0.068 s per image, which can meet the real-time of rail defects detection.

**Discussion:**

Comparing the improved method with the mainstream target detection algorithms such as Faster RCNN, SSD, YOLOv3 and other algorithms, the improved YOLOv4 has excellent comprehensive performance for rail defects detection, the improved YOLOv4 model obviously better than several others in *P*_*r*_, *R*_*c*_, and F1 value, and can be well-applied to rail defect detection projects.

## 1. Introduction

With the development of rail network layout and the rapid development of high speed rail technology, the importance of rail quality to train safety is becoming more and more obvious. According to the relevant safety statistics, the train safety accidents caused by rail surface defects account for about 30% of all accidents (Popović et al., 2022). Therefore, to ensure the security of traffic, accurate and dynamic detection of rail surface defects has become an urgent problem for railway development, and has important practical application value and research significance.

Due to the influence of rail manufacturing process, or by the wheel rail extrusion, impact, wear and other contact stress and natural weathering, its health status and quality deteriorate continuously, thus forming cracks, scars, wear, peeling, and other defects on the surface, with the passage of time, these defects will further deteriorate the rail surface quality, which may cause major railroad safety accidents. Therefore, the diversity and dynamics of rail defects bring great challenges to rail inspection technology.

The main rail defects detection methods include ultrasonic method, eddy current method, magnetic particle method, etc. (Zhao, 2021). The traditional detection methods need to rely on manual operation, time-consuming, labor-intensive, low efficiency, while it will bring unknown safety hazards to the inspectors.

Machine vision has been paid more and more attention by researchers with the benefits of fast speed, high precision and reliability, and many algorithms for surface defects detection have been generated. Faghih-Roohi et al. (2016) designed 3-layer convolution + maximum pooling layer to improve the speed of defects detection, and the accuracy of rail defects recognition can reach 92.00%, but the method only defects are detected and no classification is performed. Yuan et al. (2016) used the Otsu method to improve it by weighting the target variance of Otsu with the probability of occurrence of the target as the weight, so that the segmentation threshold close to the left edge of the single-mode histogram and the valley of the bimodal histogram, and the defects detection rate reach 93%, but the image segmentation algorithm cannot reach the real-time requirements. Shang et al. (2018) used a convolutional neural network (CNN) based on Inception-v3 to distinguish between normal and defective rail images. The model has a simple structure and faster processing speed, achieving a recognition accuracy of 92.08%, but the method is mainly effective for the detection of scar defects. Wang et al. (2018); Ni et al. (2021), and Ghafoor et al. (2022) analyzed the image features of rail defects, removed interference noise by image filtering, and then trained the model to improve the detection of surface defects, but the image enhancement algorithm is not universal and the image processing is time-consuming. Han et al. (2021) presented a multi-level feature fusion model for rail surface defects detection, which fuses the image features of different receptive field of multiple levels for target detection and enhances the accuracy of detection results and decreases the missing detection rate of small area defects, but the method detects too few types of defects and is not applicable to the detection of multiple complex defects of the rail. In summary, the above research is more concerned with the detection of defects, no classification recognition of defects, and there are problems such as image recognition methods are not universal, the speed of image processing cannot meet the defects detection of rail.

Therefore, according to the typical defect characteristics and defect types of rail, the defects are classified into four types of scars, peeling, wear and cracks, and a visual detection method combining image enhancement and deep learning is used to detect, identify and classify these four types of defects. In terms of the image processing, the captured images are firstly extracted from the rail region, then the defects edge information is enhanced with the improved Retinex algorithm, then the background modeling difference method is used to remove the background interference, and finally the defects are extracted with the adaptive thresholding. The improved Retinex algorithm and the background modeling difference method are more parameterized, and the effect on the detection speed of defects is not significant. In terms of deep learning, the Res2Net structure and attention mechanism are introduced to enhance feature extraction and improve the YOLOv4 network structure to enhance the detection rate of small-sized defects. The improved model enhances the accuracy of the four typical defects on the rail surface and ensures the detection speed.

## 2. Image enhancement algorithm for rail defects

**FIGURE 1:**
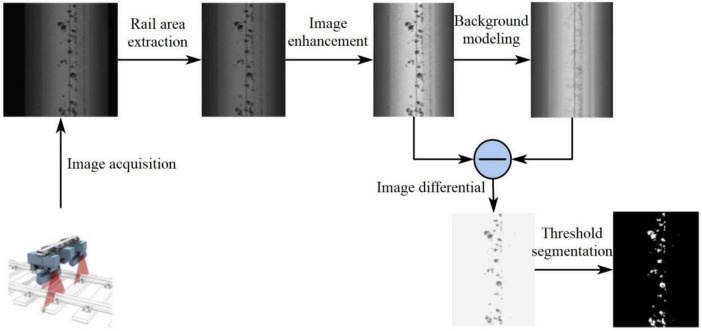
Defects detection algorithm based on background difference method.

The rail surface defects are highly susceptible to interference from lighting changes and textured backgrounds in the process of acquisition, making defects detection and recognition very difficult. To make the rail defects can be better detected and classified, the rail defects images are enhanced from four steps of rail region extraction, defects edge enhancement, background modeling difference and threshold segmentation, and the processing flow is shown in Figure 1, which solves the influence of unfavorable factors during rail surface defects segmentation.

### 2.1. Rail region extraction

To reduce the influence of textured backgrounds on rail defects detection, the column histogram minimum method (Xu et al., 2022) is first used to segment the target rail region from the original image. The steps of the column histogram algorithm are as follows:

(1) Calculate the sum of grayscale values for each column Si.
(2) Search for the minimum value min of (S_*i* + *d*_-th) at fixed rail width intervals d.
(3) The i-th column corresponding to the minimum value min is the leftmost position of the corresponding rail.
(4) The position of the rightmost rail is the (i+d)-th column.

### 2.2. Improved Retinex image enhancement algorithm

Due to environmental interference, the captured rail image has low contrast, which affects the extraction of image defect features. In addition, the two defects, wear and crack, are similar to the background, and the texture features are not obvious, which will bring great challenges to the feature extraction of the image. Therefore, the image needs to be processed to enhance the contrast of the edge contour, which helps the segmentation of this image.

Retinex is an adaptive image enhancement method (Yu et al., 2017). The theory states that the brightness of an object depends on the ambient light and the reflection of the surface of the object on the light. The reflective component is the essence of the object. The object image can be recovered by simply removing the irradiated component. The Multi-Scale Retinex (MSR) (Zhu et al., 2021a) can achieve better results by adding a weighted average of multiple scales, and its expressions are as follows:


(1) $$R_{M\,S\,R}\,\left(x,y\right)=\sum_{n=1}^{N}W_{n}\,\left\{\log\left[I\,\left(x,y\right)\right]-\log\left[I\,\left(x,y\right)\cdot G_{n}\,\left(x,y\right)\right]\right\}$$

Where, N is the total number of scales, generally taken as 3, Wn for the scale coefficient, and meet $\sum_{\text{n}=1}^{\text{N}}{\text{W}}_{\text{n}}=1$, said the number of scales for the Gaussian function. G_n_(x,y) represents the Gaussian amplifier model with the number of scales.

The MSR algorithm uses a linear quantization approach, and the processed data are widely distributed, which will show serious bifurcation and generally make it difficult to obtain satisfactory results. To enhance the edge information of rail defects, the MSR algorithm is improved from the way of quantization. The mean value and mean squared deviation are introduced, and then a parameter controlling the image dynamics is added to realize the contrast adjustment to solve the problem of serious two-level differentiation of the data and thus the unsatisfactory image enhancement effect, with the following equation.


(2) $$R\,\left(x,y\right)=\frac{255}{2}\,\left(1+\frac{\log\left[R_{M\,S\,R}\,\left(x,y\right)-\mu \right]}{D\times M\,S\,E}\right)$$

**FIGURE 2:**
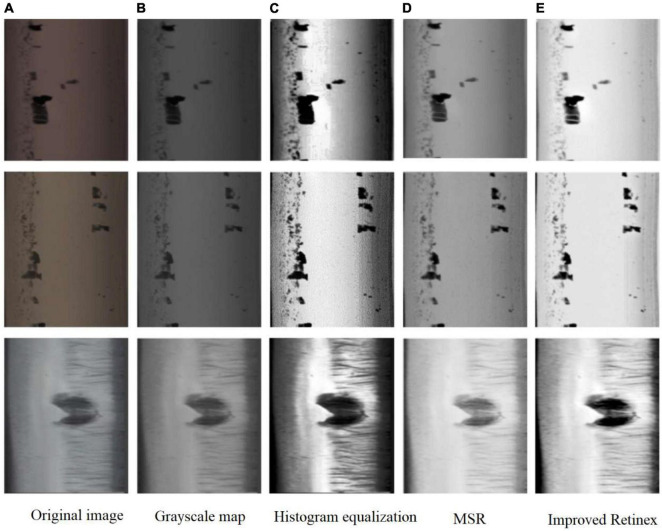
Comparison chart of the effect of image enhancement algorithm. **(A)** Original image. **(B)** Grayscale map. **(C)** Histogram equalization. **(D)** MSR. **(E)** Improved Retinex.

Where D is the dynamic adjustment parameter of the image, the value of D is inversely proportional to the contrast of the image, and μ, MSE are the mean and mean squared deviation of the number of channels of R, G, B in log [R_*MSE*_ (x, y)], respectively, and Value is the value of log [R_*MSE*_ (x, y)]. After the experiment, the best effect is obtained when the scale number is 3 and D is 2.5 (Figure 2).

The results show that the improved Retinex has stronger contrast and more prominent defect edges information than MSR, and less noise than histogram equalization. If the results of MSR are quantified directly, the overall darker images are obtained, which is due to the smaller data range of the original values after logarithmic processing and the small differences between channels, and the linear quantization is much smoother than the logarithmic curve, so the overall effect is darker and the edge information is easily lost. Proposed in this paper achieves good results by changing the quantization of the mean and mean squared deviation to strengthen the defect edges. The average Peak Signal to Noise Ratio (PSNR) per image is calculated to be 15.40, which is a very significant improvement in image quality and is very suitable for the processing of orbital defect images.

### 2.3. Background difference segmentation algorithm for surface defects

To segment the rail defects from the background image, the defects segmentation based on background difference algorithm is proposed, the idea of background difference method is the process of subtracting the background from the current image so as to get the defects. The background image is obtained by learning the rail video sequence, and the method of extracting the motion foreground in the video sequence based on background difference is mainly divided into three steps (Chel et al., 2020): background modeling, foreground detection, and background update. Among them, the mean method is the simplest in background modeling (Piccardi, 2004), which can quickly and effectively segment moving targets in static scenes with high real-time performance.

Since single image defects segmentation cannot learn the background model from the video sequence, the background difference method in video surveillance cannot be directly used for rail surface defects segmentation. Considering the feature of small variation range of gray value along the rail direction of the image and the real-time requirement, rail surface defects segmentation algorithm based on the mean background difference is proposed.

#### 2.3.1. Background modeling

Define the direction perpendicular to the rail as the x−axis and the rail direction as the y−axis. Calculate the mean value of each column of the image according to the feature of small change of the image along the y−axis, and modeling the background image.


(3) $$I_{m}\,\left(x\right)=m\,e\,a\,n\,\left(I_{y}\,\left(x\right)\right)$$

Where *I*_*m*_(*x*) denotes the x-th column image background modeling and *mean*(*I*_*y*_(*x*)) is the mean value function.

The algorithm implements static single-image background modeling, and the processing speed is not affected due to the simplicity of modeling, and the background is maximally close to the original image.

#### 2.3.2. Background subtraction

To highlight defects and diminish the effects of illumination variations and reflection unevenness, and subtract the rail image from the background image to get the difference image.


(4) $$\Delta \,I\,\left(x,y\right)=I_{0}\,\left(x,y\right)-I_{m}\,\left(x,y\right),\forall \left(x,y\right)$$

where *I*_0_(*x*,*y*) is the original image and *I*_*m*_(*x*,*y*) is the modeled background image.

#### 2.3.3. Adaptive thresholding segmentation

To segment the defective regions in the differential images, Niblack thresholds are defined (Zhou et al., 2013).


(5) $$t\,h=\mu_{\Delta \,I}+C\cdot \delta_{\Delta \,I}$$

Where μ_Δ*I*_ and δ_Δ*I*_ are the mean and variance of Δ*I*, respectively, and the control factor *C* is a constant. Following Chebyshev⁢s′ formulas, ratio of data with more than *C* times the Standard Deviation (SD) from the mean is at most 1/*C*^2^ in any dataset. For this purpose, the value of *C* can be determined based on the ratio of the target defects to the total image. Since the differential image has the property of zero mean, Equation 5 can be simplified as follows.


(6) $$t\,h=C\cdot \delta_{\Delta \,I}$$

**FIGURE 3:**
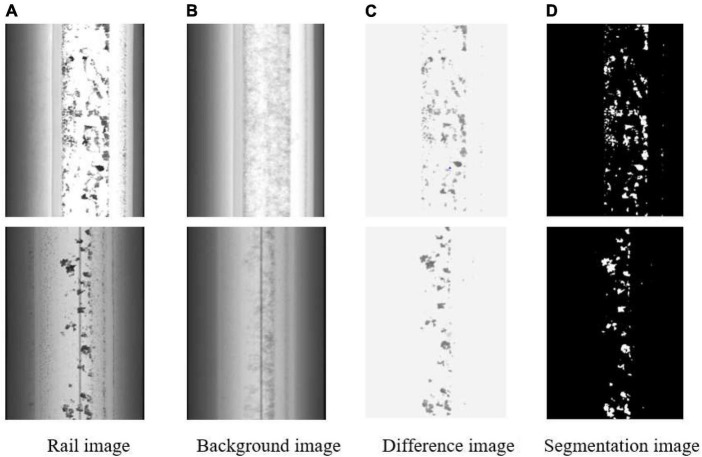
Splitting effect of the rail images. **(A)** Rail image. **(B)** Background image. **(C)** Difference image. **(D)** Segmentation image.

After experiments, the segmentation effect is best when *C* = 3. The method can segment the defects well according to the obtained threshold t*h* for the image. The processed ones are shown in Figure 3.

## 3. Improved YOLOv4 model for rail defects detection

YOLOv4 has a high performance in recognizing large and medium-sized, significantly separated targets (Bochkovskiy et al., 2020), but the detection accuracy is not high for small-sized targets and targets with small background differences. In the dataset used in this paper, most of the Scar and Peeling defects are small in size, and the foreground background differences of Wear and crack defects are small, which are not ideal for the recognition of defects directly with the YOLOv4 network. Accordingly, the network structure and feature extraction aspects are optimized based on the YOLOv4 network to adapt it to the detection and recognition of orbital defects.

### 3.1. Rail defects feature extraction method

#### 3.1.1. Introduction of Res2Net

Aiming at the problem of small size and little detail information of rail defects, Res2Net structure and attention mechanism are introduced to enhance the feature extraction of defects.

**FIGURE 4:**
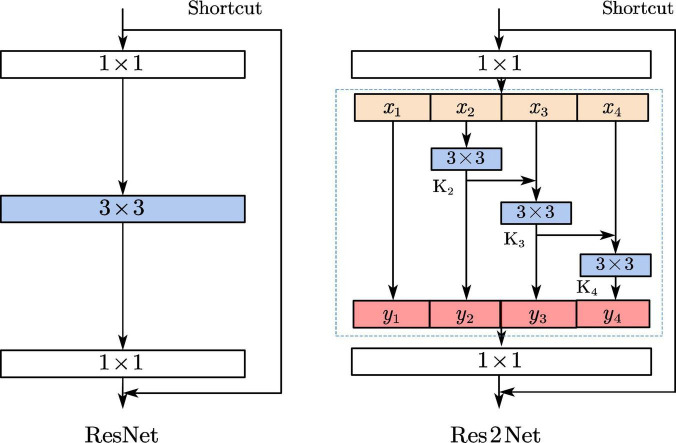
Structure of ResNet and Res2Net.

The ResNet residual blocks in the YOLOv4 network structure are replaced with the Res2Net structure, as shown in Figure 4. This structure not only increases the receptive field of each network layer, but also enhances the ability of multi-size feature extraction and enables effective detection of small-size defects.

In the Res2Net structure, each output can increase the receptive field, where y2 can get a 3x3 receptive field, can y3 get a 5x5 receptive field, and y4 can get a larger 7x7 receptive field, so each Res2Net can obtain a combination of features with different receptive field sizes. Thus, the structure can both increase the receptive field of each network layer, and fuse multi-scale features. It is very effective for the small-sized targets (Gao et al., 2021).

#### 3.1.2. CBAM attention mechanism

**FIGURE 5:**
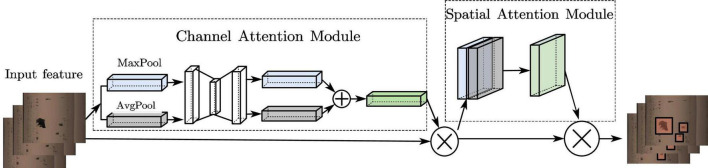
CBAM overall structure diagram.

To enhance the attention to the effective feature information and to improve the region weight of rail defects, an attention mechanism is added to the model. Convolutional Block Attention Module (CBAM) (Woo et al., 2018) is a lightweight attention module based on CNN, and is shown in Figure 5. It integrates the Channel Attention Module (CAM) (Ilyas et al., 2021) and the Spatial Attention Module (SAM) (Hu et al., 2020) to generate the corresponding feature map mapping to increase the weight of the defects region in the feature map, which in turn makes the model pay more focus to the features of the defects location and reduces the influence of background and uneven spatial distribution on the detection of rail defects.

##### 3.1.2.1. In channel attention

The rail defect features are max-pooled and average-pooled, respectively, to obtain 2 “1×1×C″ channel descriptions, and then they are sent into a 2-layer shared fully-connected layer, and the two output features are summed up to obtain a weight coefficient after the activation function, and eventually the new features multiplied by the weight coefficients and the original features are used as input for the SAM.

##### 3.1.2.2. In SAM

Global average pooling as well as global maximum pooling operations are performed on channels to produce 2 feature maps represent different information. After merging them, feature fusion is proceeded by 7×7 convolution with a larger receptive field, and lastly the operation is used to generate a weight map, which is then superimposed on the original input feature map to obtain a final rail defects feature map.

The feature map of CBAM is the same size as the feature map of original image, only the feature elements have changed, focusing more on the edge location information of the defects image, reducing the impact of background on detection accuracy and reducing the rate of wrong and missed detection. It can help the network to extract features better and deeper, and further improve the network’s ability to learn rail defects.

### 3.2. Design of defects recognition network

#### 3.2.1. Network structure

The PANet structure used in YOLOv4 can fuse the semantic information of different feature layers and is suitable for detecting targets of different sizes. However, the number of rail surface defects is high and the proportion of pixels in the image is low, and the original PANet structure still lacks effective detection for tiny defect targets. Therefore, on the basis of the original feature layer, Continue to fuse shallow and deep features to increase the feature detection scale and form a new feature detection layer.

**FIGURE 6:**
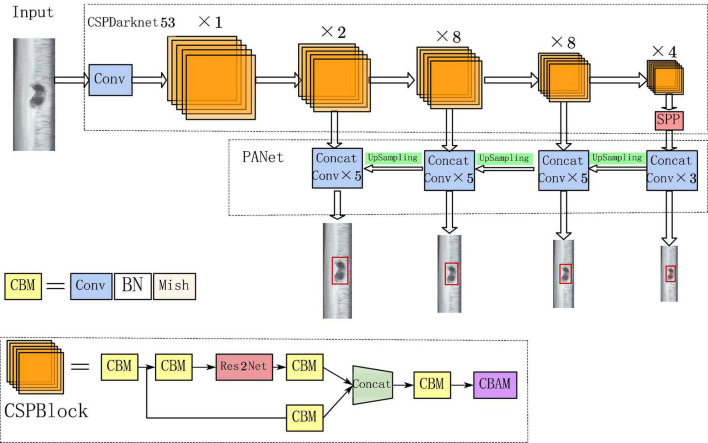
Improved YOLOv4 network structure diagram.

Adding new feature detection layers leads to an increase in the number of network structure parameters, and the bottom-up path enhancement structure contributes less to the detection of small area defects. Therefore, the bottom-up path enhancement structure in PANet is removed in order to reduce parameter redundancy and ensure sufficient detection speed. Meanwhile, to help the network extract features better and deeper, the residual structure in the CSPBlock block is replaced with the Res2Net structure; to further improve the network’s ability to learn rail defects, the CBAM structure is added to the CSPBlock block. The improved PANet structure is shown in Figure 6. The improved structure not only inherits the feature fusion effect of the original structure, but also can obtain more shallow features while reducing the network parameters, so the feature extraction effect of small area defects of the rail is better.

#### 3.2.2. Anchor frame clustering

Since a new feature detection layer is added, the number and size of anchor frames are not suitable for this network, so it needs to be re-clustered. K-means is used in YOLOv4 network, and the clustering effect is largely determined by selecting the initial cluster center. To ensure a relatively good clustering effect, K-means++ is adopted to re-cluster the anchor frames. The method of clustering is as follows:

1. Randomly select a sample from the rail defects dataset as the initial cluster center *v*_*j*_.
2. Secondly, calculate the distance between each sample *x*_*i*_ and *v*_*j*_ in the dataset and select the shortest of them.
3. Then calculate the probability of each data sample being selected as the next clustering center, and select the sample with the greatest probability distance as the new clustering center.
4. Repeat steps 2 and 3 until all k clustering centers have been identified.
5. Cluster the *k* initialized cluster centers obtained, assign each sample to the cluster center with the smallest distance from each other, and update the cluster centers, and repeat the step until the cluster centers unchanged.

The clustering results are shown in Table 1.

**TABLE 1:** *A priori* box clustering results.

Clustering algorithm	Prior box
Entry 1K-means	(12,14), (15,23), (17,44), (40,26), (41,93), (48,49), (33,151), (63,78), (85,45), (61,125), (74,223), (134,82)
K-means++	(11,13), (16,21), (18,42), (37,27), (51,51), (78,41), (33,152), (40,85), (65,80), (60,123), (71,222), (118,77)

From Table 1, it can see that most of the anchor frames are very different from each other, except for the first three groups of anchor frames, which do not vary much. Compared with k-means randomly selecting the cluster center, k-means++ selects the cluster center by the idea of “the farther the cluster center are from each other, the better,” which converges the data faster and achieves good results while reducing the computation time.

## 4. Experiment and analysis

Evaluation metrics for training and performance are first established, and then the current mainstream deep learning-based target detection algorithms are compared with the algorithms of this paper in terms of accuracy and speed metrics. The computer configuration is a 64-bit Windows 10 system with 32G of RAM, CPU model i9-10980XE, and GPU model is RTX3090. In the training process, the bitch_size is set to 16, the initial learning rate is 0.001, the learning rate is decayed, the final learning rate is 0.00001, and iterations is set in 1,000. A 416 × 416 resolution input is taken for training, the detection threshold is set to 0.5, and the Dropout method is used to prevent overfitting.

### 4.1. Dataset and evaluation index

**FIGURE 7:**
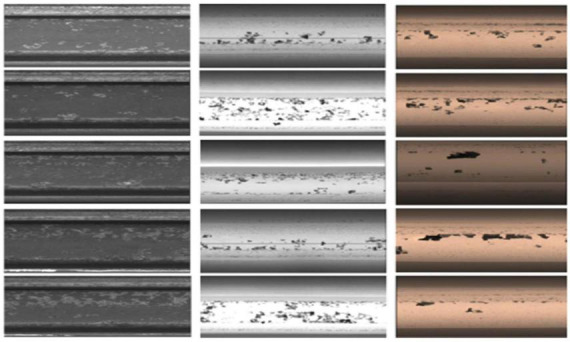
Image samples collected by three different types of line-scan CCDs. The figure has been licensed by Gan et al. (2017).

The experimental dataset were obtained from Rail beam factory of Panzhihua Iron and Steel (Group) Company and network datasets, where the self-acquired dataset were used for training and the RSDDs (Gan et al., 2017) network datasets were used for validation. For the image acquisition experiments, color/grayscale images of heavy rails of 60 kg/m were obtained using three different types of line-scan CCD cameras, and a total of 2,124 images of rails with high imaging quality were selected, of which 956 were defective, and image samples are shown are shown in Figure 7.

**FIGURE 8:**
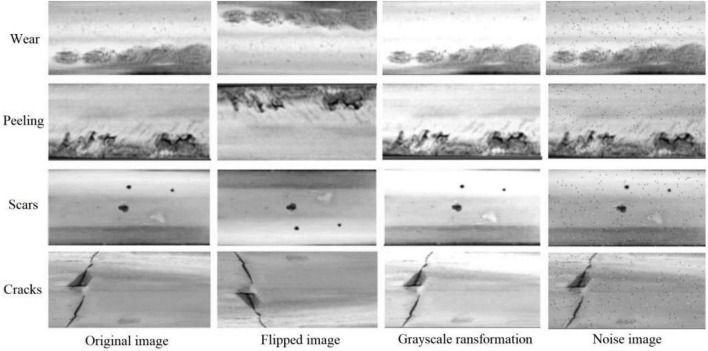
Expansion of the dataset.

To unify the experimental dataset, all acquired images are first segmented on the rail surface, and then the images are resized to 400*800 pixels, and finally the dataset is expanded by flip transform, brightness transform, random cropping, geometric scaling, etc., 4,000 images of rail surface defects dataset are generated, including 1,000 images each of cracks, scars, wear and peeling. Randomly select 80% as the training set, and 20% as the test set. Figure 8 shows the typical samples of the four defects and their expansions.

This paper introduces four evaluation indexes: Recall Rate (*R*_*c*_ or *R*), Precision Rate (*P*_*r*_ or *P*), F1 Value and Average Inspection Time. Rail surface damage detection is related to the safety of railroad transport, and both *R* and *P* indexes are particularly important, while F1 value can visualize the importance of *R* and *P*.


(7) $$R=\frac{T\,P}{T\,P+T\,N}$$

(8) $$P=\frac{T\,P}{T\,P+F\,P}$$

(9) $$F\,1=\frac{2\,P\,R}{P+R}$$

Where: *TP*: Positive samples predicted to be positive class, *FP*: Negative samples predicted to be positive class, *TN*: Negative samples predicted to be negative class.

### 4.2. Algorithm performance analysis

Training and test experiments were conducted on several detection algorithms [Faster R-CNN (Sekar and Perumal, 2021), SSD (Liu et al., 2016), YOLOv3 (Redmon and Farhadi, 2021), YOLOv4 (Bochkovskiy et al., 2020), YOLOv5 (Zhu et al., 2021b), YOLOv6 (Li et al., 2022)] and the improved algorithms in this paper, and after the network training and parameter tuning, the network convergence, and then the data results were tallied according to the evaluation metrics.

**FIGURE 9:**
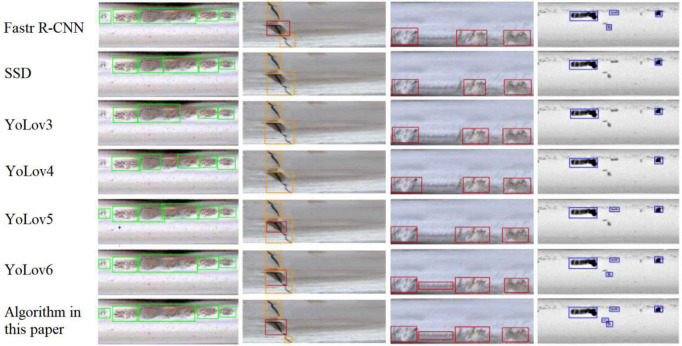
Comparison chart of the effect of detection.

The data in Table 2 show that the detection algorithm of this paper has the highest *R*_*c*_ and *P*_*r*_ for four defects: cracks, scars, wear, and peeling. Relative to other mainstream algorithms, the improved YOLOv4 algorithm has an F1 value that is 2.2% higher than Faster R-CNN, 8.1% higher than SSD, 8.2% higher than YOLOv3, 5.2% higher than YOLOv4, 4.0% higher than YOLOv5, and 1.1% higher than YOLOv6. All 3 metrics are better than other mainstream detection algorithms. Compared with the original YOLOv4 network, the accuracy of the improved network reaches 94.8% for cracks, 6.4% higher than before the improvement; 94.0% for scars, 7.9% higher than before the improvement; 89.7% for wear, 5.6% higher than before the improvement; and 92.2% for spalling, 3.1% higher than before the improvement. The accuracy rates of the four typical defects are 1.2, 1.6, 1.0, and 1.8% higher than YOLOv6, respectively, which is a significant improvement. In addition, although this algorithm increases the detection layer and adds the attention mechanism resulting in increased parameters, the removal of the bottom-up path structure in the PANet reduces a large number of parameters, and the image pre-processing of the background difference method is concise and effective. The average detection time per image is 0.068 s (68 ms), which is very close to that of YOLOv4, YOLOv5, and YOLOv6, and can meet the system real-time requirements while ensuring the effect of rail defects detection. Mapping the inspection results back to the original image, the effect comparison chart is shown in Figure 9, where the green box is for wear defects, the orange box is for crack defects, the red box is for peeling defects, and the blue box is for scar defects.

**TABLE 2:** Comparison of the detection performance of different algorithms for the self-collected dataset.

Detection algorithm	Cracks		Scars		Wear		Peeling		F1	T/ms
	**P/%**	**R/%**	**P/%**	**R/%**	**P/%**	**R/%**	**P/%**	**R/%**		
Faster R-CNN	91.2	93.1	89.8	90.3	88.5	86.9	90.7	91.6	0.903	41.2
SSD	86.3	88.6	84.1	87.2	80.7	77.9	84	86.3	0.844	82.0
YOLOv3	85.8	87.5	84.3	85.8	79.2	76.5	86.9	88.2	0.843	46.0
YOLOV4	88.4	90.4	86.1	85.3	84.1	83.9	89.1	91.2	0.873	55.0
YOLOv5	90.1	91.7	88.6	88.9	85.3	84.1	89.2	90.5	0.885	49.0
YOLOv6	93.6	92.3	92.4	92.8	88.7	89.2	90.4	91.7	0.914	40.0
Algorithm in this paper	94.8	93.7	94.0	93.6	89.7	88.4	92.2	93.6	0.925	68.0

**FIGURE 10:**
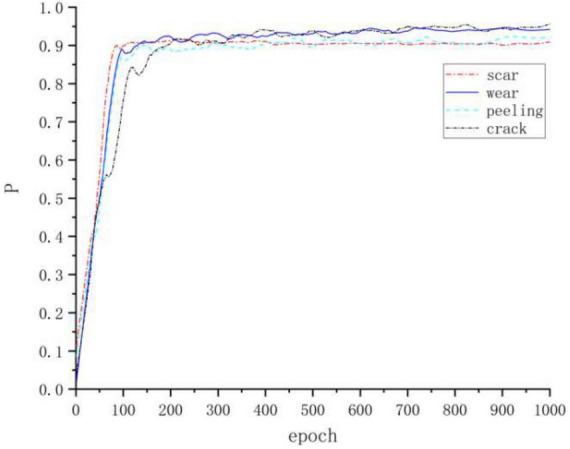
Detection accuracy of the four defects.

From Figure 9, this algorithm can recognize defects of small size and defects with small background differences very well, and the recognition effects are all better than other mainstream algorithms, and Figure 10 shows the accuracy of four kinds of defects.

To continue to verify the effectiveness of this algorithm, the algorithm is tested on the publicly available dataset RSDDs, comparing the method of this paper with improved Cascade R-CNN proposed by Luo et al. (2021), improved YOLOv5 proposed by Guo et al. (2022) and multi-layer feature fusion network proposed by Han et al. (2021), the defects detection accuracy and the average detection time of a single image are shown in Table 3.

**TABLE 3:** Comparison of detection performance of different algorithms for RSDDs dataset.

Literature sources	Network structure	AP/%	T/ms
Luo et al. (2021)	Improved cascade R-CNN	98.75	146.3
Guo et al. (2022)	Improved YOLOv5	91.80	54.8
Han et al. (2021)	Multi-layer feature fusion network	96.72	59.8
Algorithms in this paper	Improved YOLOv4	98.96	68.0

The accuracy of this algorithm for defect detection on the RSDDs rail dataset reaches 98.96%, all of which are better than the methods used by the other three. The average detection time per image is 68 ms, which is significantly better than Luo’s method and very close to Li and Han’s methods, and fully satisfies the real-time performance of rail defects detection. The results show that this method is more suitable for performing the task of rail surface defects detection.

### 4.3. Ablation experiments

The algorithm uses several improved strategies based on YOLOv4, and to verify its effectiveness, ablation experiments were designed for comparative analysis.

**FIGURE 11:**
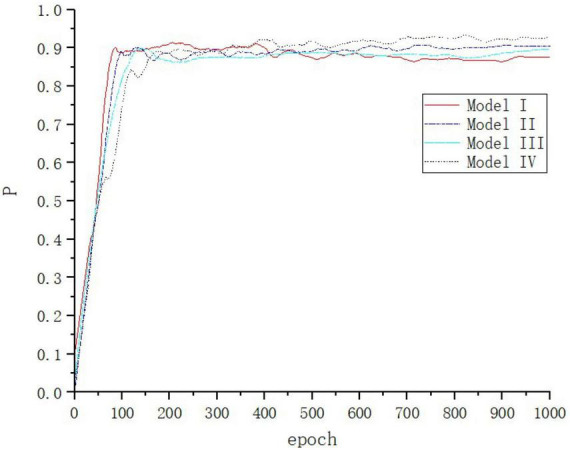
Accuracy change curve of each model in ablation experiment.

Model I: YOLOv4 network. Model II: The model obtained by replacing the Residual Block structure in the feature extraction part of YOLOv4 with the Res2Net module, and then adding the CBAM attention mechanism. Model III: Adding the detection layer and removing the top-down structure in PANet. Model IV is the model of this paper. Each network model is trained for 1,000 cycles (Figure 11).

In this figure, the loss values of each network model in the ablation experiments decrease rapidly within the first 50 iterations of the training process, and then gradually converge.

As seen in Table 4, Model II makes improvements to feature extraction, and increasing the weight of defects location and increasing the perceptual field to better extract the small defect features of the rails, with a 2.75% improvement in *P*_*r*_, 1.99% improvement in *R*_*c*_, and 2.40% improvement in F1 value over the YOLOV4 network, effectively improving the detection performance of small size defects. The model III network structure performs multi-scale feature fusion to enhance the accuracy of defects localization, which improves *P*_*r*_ by 1.41%, *R*_*c*_ by 1.74%, and F1 value by 1.60% over the YOLOV4 network, but the detection time of a single image increases by 8 ms, which is due to the increase of detection layers, resulting in the calculation of a large number of additional parameters. The fusion of the above two improved methods into the benchmark network at the same time can further improve the accuracy of rail defects localization and identification, which improves *P*_*r*_ by 4.98%, *R*_*c*_ by 5.40%, and value by 5.20% over the YOLOV4 network. This verifies the validity of the improved method for rail surface defects detection.

**TABLE 4:** Results of ablation experiments.

Network model	P_r_/%	R_c_/%	*F1* value	T/m*s*
Model I	87.70	86.93	0.873	55
Model II	90.45	88.92	0.897	59
Model III	89.11	88.67	0.889	63
Model IV	92.68	92.33	0.925	68

## 5. Discussion

For the problem of small defects size and complex background of rail. The detection algorithm for rail surface defects is proposed. The improved YOLOv4 defects detection algorithm not only inherits the feature fusion effect of the original structure, but also can obtain more shallow features while reducing the network parameters and improving the feature extraction capability of small targets. The average processing speed of a single image is only 13 ms higher than YOLOv4, which is also very close to the detection speed of YOLOv6. Efficient and accurate detection of rail defects is achieved, where the recognition accuracy of 4 defects, namely, cracks, scars, wear and peeling, reaches 94.8, 94.0, 89.7, and 92.2%, respectively. Their *P*_*r*_, *R*_*c*_ and F1 values are higher than other mainstream target detection algorithms. The detection algorithm ensures high detection accuracy while guaranteeing detection speed, and is more suitable for performing rail surface defect detection tasks.

## Data availability statement

The original contributions presented in this study are included in this article/supplementary material, further inquiries can be directed to the corresponding authors.

## Author contributions

ZM organized the database. RC performed the statistical analysis and wrote the first draft of the manuscript. All authors contributed to conception and design of the study, wrote sections of the manuscript, revised the manuscript, and read and approved the submitted version.
